# Decentralizing evidence-based decision-making in resource limited setting: A case of SNNP region, Ethiopia

**DOI:** 10.1371/journal.pone.0236637

**Published:** 2020-07-30

**Authors:** Misganu Endriyas, Abraham Alano, Emebet Mekonnen, Aknaw Kawza, Fisha Lemango

**Affiliations:** SNNPR Health Bureau, Hawassa, Ethiopia; Technion - Israel Institute of Technology, ISRAEL

## Abstract

**Background:**

Access to and the use of accurate, valid, reliable, timely, relevant, legible and complete information is vital for safe and reliable healthcare. Though the study area has been implementing standardized Health Management Information System (HMIS), there was a need for information on how well structures were utilizing information and this study was designed to assess HMIS data utilization.

**Methods:**

Facility based retrospective study was conducted in Southern Nations Nationalities and People’s Region (SNNPR) in April, 2017. We included data from 163 sample facilities. Data use was evaluated by reviewing eight items from performance monitoring system that included activities from problem identification to monitoring of proposed action plans. Each item reviewed was recoded to yes or no and summed to judge overall performance.

**Results:**

About half (52%) of woredas, 26.2% health centers (HCs), 25% hospitals and 6.2% health posts (HPs) reviewed their performance monthly but only 20% woredas, 6.2% HCs, 1.5% HPs and no hospital prepared action plans after reviewing performance. Summary of 8 items assessed showed that majority of facilities (87.5% hospitals, 81.5% HPs and 70.8% HCs) were poor in data utilization.

**Conclusions:**

Only about half of woredas and below one-fifth of health facilities were utilizing HMIS data and a lot to move to catch-up country’s information revolution plan. Lower health care systems should be supported in evidence-based decision-making and progress should be monitored routinely quantitatively and/or qualitatively.

## Background

Health management information system (HMIS) is a process of recording, storing, retrieving and processing health data for decision-making [1–4]. Access to and the use of accurate, valid, reliable, timely, relevant, legible and complete information is vital for safe and reliable healthcare [5]. Improving HMIS helps decision makers to detect and control health problems, monitor and evaluate performance, and promote equity, empowering individuals and communities with health-related information and strengthening the evidence base for effective health policies [4, 6–12].

Globally, significant human and financial resources have been invested in the collection of data from different sources, including facility and community based sources and knowledge and understanding of its role have improved. However, this information is often not used by key stakeholders and as a result, many health programs fail to fully connect evidence to decisions needed to respond to priority needs [13] and is still very weak in most low- and middle-income countries (LMICs) [14].

The Paris Declaration and initiatives such as the Health Metrics Network, the Millennium Development Goals, and the Sustainable Development Goals have prompted LMICs to make the development of well-performing HMIS a high priority. WHO and the MEASURE Evaluation project have supported many LMICs in Africa and Asia and improvements have been documented in HMIS [14].

Ethiopia has been implementing HMIS at all health service delivery system levels for ensuring information use for evidence-based health planning and decision-making [15]. Ethiopia also revised HMIS reform focusing on rationalizing and standardizing indicators, data collection and reporting forms and procedures, and institutionalizing HMIS data quality assurance and information use mechanisms [16]. In addition, information revolution is one of the four transformation agendas in the current health sector transformation plan which focuses on addressing quality and equitable distribution of health service delivery for all [17].

In Southern Nations Nationalities and People’s Region (SNNPR), regional health bureau has been implementing electronic health management information system (eHMIS) and electronic integrated disease surveillance report (eIDSR) in collaboration with partners working in the area of HMIS. As part of data quality assurance, performance review teams are organized from health bureau to health facilities and regularly review performance and there is structure of reporting to higher level and giving feedback to lower structures. But designing and implementing the information system may not realize the information utilization [18]. So, there was a need for information on how well structures were utilizing information and this study was designed to assess HMIS data utilization in SNNPR Ethiopia.

## Methods

Facility based retrospective document review of HMIS performance monitoring system was conducted in SNNPR Ethiopia in 2017. Administratively the region was divided into 14 zones, one city administration and four special woreda [19]. In the region, zones are divided in to woredas (districts). And special woreda is an administrative structure equivalent to district but not contained in zones and directly accountable to region. City administration is the capital of the region and divided to sub-cities. All administrative structures are then finally divided in to “Kebele”, the smallest administrative structure. So, the administrative structure from higher to lower is region, zone, woreda and kebele or region, special woreda and kebele and city administration is structured in a way region, city administration, sub-city and kebele.

In 2017, the region had 57 hospitals of all type (specialized, general and primary), 736 health centers and 3865 community level health posts. In Ethiopia, health post is community level (Kebele level) health services delivery point providing set of services mainly preventive and community services. Services are provided by usually two health extension workers who have got basic trainings after completion of high school and recruited by government. Health center is health facility that delivers standard primary health care, both preventive and curative services and supports on average five catchment health posts.

The study included assessment of data quality and utilization in sample of 163 institutions of different types: 65 health posts, 65 health centers, 8 hospitals and 25 woreda health offices. The sample size was estimated using sample size formula for facility survey and allocated to zones and special woredas proportionally considering number of functional facilities. Even though number of community level health post was high, we assessed one health post from health posts reporting to selected health center. Facilities were selected using multi-stage sampling. First, woredas were selected and at second level, facilities were selected using simple random sampling. For data quality, data accuracy of selected data elements from routine HMIS reports was assessed. To do this, recounts in registers (or tally in case of health posts) and reports over the same period were compared and difference was reported using verification factor. The detail about the region and procedure is available in data accuracy assessment part [20]

We used data from October to December, 2016 as it was latest completed quarter of budget year. BSc holder nurses and health officers were recruited to collect data considering prior experience in HMIS and data collection and were trained (with field-test) for three days on tools and procedures including ethics. Daily supervision was done by supervisors who had MSc and experience in HMIS and data collection. All collected data were examined for completeness and consistency of data and checklists that were incomplete or inconsistent were re-administered. Data were entered, cleaned and analyzed using IBM SPSS for Windows version-20. Descriptive statistics were done to describe variables using actual numbers and percentages.

The MEASURE evaluation guide suggested steps to use routine data to improve programs byproviding guidance in linking questions of interest to program managers and providers to existing data; analyzing, graphing, and interpreting data; and continuing to monitor key indicators to inform improvements. The steps indicated are identifying questions of interest, prioritizing interests, identifying data sources, transforming data, interpreting information, designing solutions and taking actions and monitoring performances [21].

To assess utilization of data, we reviewed activities of performance review team /PRT/. This included problem identification, problem prioritization, preparing action plan, monitoring implementation of action plan, data visualization and assessing data quality. In the study area, PRT is a team comprising decision-makers and technical personnel that review performance on monthly basis, prepare action plans, communicate it to concerned bodies and monitor implementation of action plans. To assess these issues and judge overall data utilization, we summed performance of 8 items: availability of minute of PRT, minute addressing high and low performances, problem prioritization (action plan), actions (activities report based on action plan), data presentations (up-to-date infant immunization monitoring and up-to-date malaria monitoring chart if there was malaria case report) and data quality assessment.

Availability of minute written during PRT meeting was assessed and was examined if the discussions covered high and low performances to identify lessons and gaps for planning. Immunization and malaria monitoring charts were assessed as part of data visualization for easy capturing. Immunization chart shows monthly and cumulative coverage of infant immunization for budget year and malaria monitoring chart shows trend of malaria incidence compared with expected threshold. Action plan shows priority activities identified to give attention towards the target, including responsible person and time frame. After preparing action plan, the plan is communicated to responsible person and implementation of action plan is followed by reviewing reports from responsible persons. Finally, data quality assessment is key component of data use by which data in hand is examined for its accuracy. To do data quality (in terms of accuracy) assessments, reports are compared with source document over the same period. Source document can be registration book or tally or reports from which aggregate was compiled depending on data elements selected.

Each observed item was recoded to yes (1) or no (0) based on its availability at time of assessment. To recode PRT performance items available or yes (1), we considered availability of two or more demonstrations in the quarter while display items like infant immunization and malaria monitoring charts were checked for being up-to-date. Finally, we categorized performance of 75% or more as good utilization, 50–74% as fair utilization and <50% as poor utilization.

Ethical clearance was obtained from SNNPR Regional Health Bureau Ethical Review Board. After getting ethical clearance, official letter was written to each study areas. Verbal consent was also obtained from each individual respondent and data handler after through explanation of the purpose, benefit, risk and confidentiality of the study. The information obtained kept anonymous and confidential.

## Results

The study covered 25 Woreda health offices, 73 health facilities (65 health centers and 8 hospitals) and 65 health posts, a total of 163 institutions from 14 zones, 1 city administration and one special woreda.

### Evidence based decision making system

As part of evidence based decision making system, items assessed were summarized in to problem identification, problem prioritization, preparing action plan, monitoring implementation of action plan, data display and assessing data quality.

#### A. Problem identification

As part of problem identification, we considered presence of organized performance review team minutes documented during review meetings and whether contents addressed high and low performance figures. All woredas, 98.5% HCs, 87.5% hospitals and 53.8% HPs could show lists of members of performance review team. Three minutes on monthly performance review were expected from each facility over three months. Table 1 shows number of minutes in quarter and whether the contents addressed high and low performance values. Half (50.8%) of HPs had no minute while only 6.2% had complete three minutes in the quarter, from which only 1.5% addressed high and low performances. Compared to facilities, woreda was performing better in that about half (52%) had complete three minutes addressing both high and low performances and only eight percent no minute in the quarter.

**Table 1:** Problem identification by public health facilities, SNNPR, 2017.

Performance review using minutes	Number of minutes	Facility types			
		Woreda No (%)	HC No (%)	Hospital No (%)	HP No (%)
Minute reported available		24 (96)	60(92.3)	8(100)	35 (53.8)
Number of minutes observed in the quarter	0	2 (8)	7(10.8)	0 (0)	33 (50.8)
	1	3 (12)	29(44.6)	6(75)	10 (15.4)
	2	7 (28)	12(18.5)	0 (0)	18 (27.7)
	3	13 (52)	17(26.2)	2(25)	4 (6.2)
Number of minutes addressing high figures	0	2 (8)	7(10.8)	0 (0)	43 (66.2)
	1	3 (12)	40(61.5)	5(62.5)	8 (12.3)
	2	7 (28)	15(23.1)	3(37.5)	13 (20.0)
	3	13 (52)	3(4.6)	0 (0)	1 (1.5)
Number of minutes addressing low figures	0	2 (8)	10(15.4)	0 (0)	41 (63.1)
	1	3 (12)	40(61.5)	6(75)	7 (10.8)
	2	7 (28)	12(18.5)	2(25)	16 (24.6)
	3	13 (52)	3 (4.6)	0 (0)	1 (1.5)

#### B. Problem prioritization

**Fig 1:**
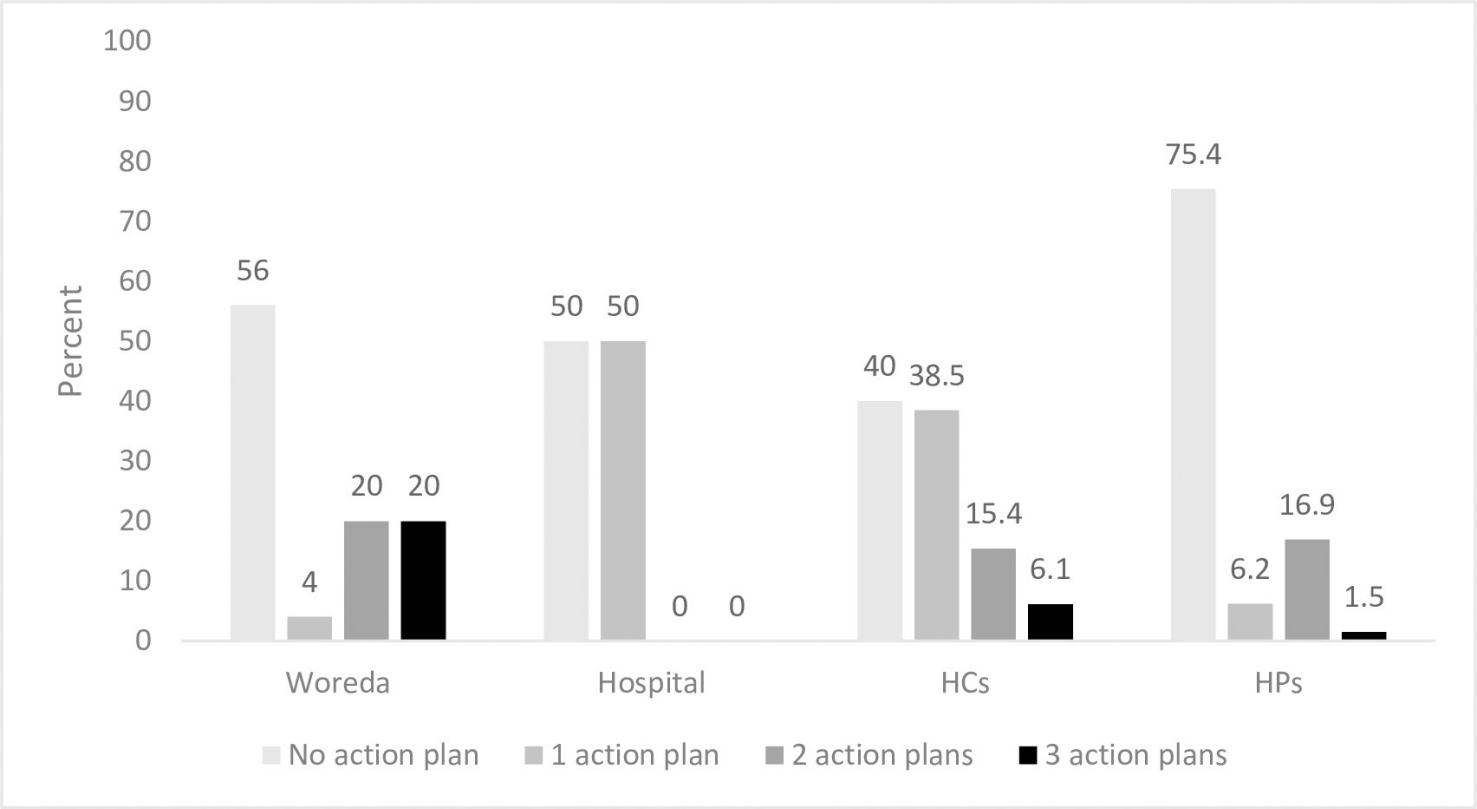
Proportion of facilities with action plans.

After reviewing performance, the performance review team is expected to plan actions to observed (identified) gaps. Even though 64% woredas, 69.2% HCs, 50% hospitals and 30.8% HPs reported that they prepare action plans, only less than half of facilities prepared action plans (Fig 1). More specifically, 20% woredas, 0% hospitals, 6.1% HCs and 1.5% HPs prepared complete three action plans in the quarter.

#### C. Action on identified gaps

After preparing action plan, the prepared action plan should be communicated to responsible person and activities performed should be monitored by performance review team for implementation of agreed action plan. Sixteen (64%) woredas, 39 (60%) HCs, 3 (37.5%) hospitals and 18 (27.7%) HPs reported that they implement action plans prepared and monitor it. The implementation of action plan was measured by reviewing minute of subsequent meeting for its content discussing implementation status of previous action plan and/or actions reported by responsible person. And 48% woredas, 52.3% HCs, 62.5% hospitals and 81.5% HPs had no data indicating implementation of action plan while only 24% woredas, 3.1% HCs, 0% hospitals and 3.1% HPs showed complete three reports on previous action plan or minutes of reviewing of previous action plan (Table 2).

**Table 2:** Preparing action plan for identified gaps by public health facilities, SNNPR, 2017.

Actions based on action plan		Facility types			
		Woreda No (%)	HC No (%)	Hospital No (%)	HP No (%)
Actions reported		16 (64)	39 (60)	3 (37.5)	18 (27.7)
Number of action plan implementation reports	0	12 (48)	34 (52.3)	5 (62.5)	53 (81.5)
	1	1 (4)	15 (23.1)	3 (37.5)	2 (3.1)
	2	6 (24)	14 (21.5)	0 (0)	8 (12.3)
	3	6 (24)	2 (3.1)	0 (0)	2 (3.1)

#### D. Data display

Data display helps concerned bodies to easily capture information on performance. In the study area, the following data are usually included during routine performance assessments among other key performance measurement indicators. Two-thirds (60%) woredas, 58.5% HCs, 50% hospitals and 36.9% HPs displayed up-to-date EPI chart (Table 3).

**Table 3:** Data display by public health facilities, SNNPR, 2017.

Data display	Response	Facility types			
		Woreda No (%)	HC No (%)	Hospital No (%)	HP No (%)
Up-to-date EPI monitoring chart	EPI monitoring chart reported	22 (88)	55 (84.6)	4 (50)	47 (72.3)
	EPI monitoring chart observed	15 (60)	38 (58.5)	4 (50)	24 (36.9)
Up-to-date malaria monitoring chart	Malaria monitoring chart reported	18 (72)	46(70.8)	3(37.5)	35 (53.8)
	Malaria monitoring chart observed	14 (56)	33(50.8)	3(37.5)	22 (33.8)
Top ten causes of morbidity*	Top ten reported		52 (80)	8 (100)	
	Top ten observed		42 (64.6)	6 (75)	

*- Top ten causes of morbidity is more appropriate at HC and hospital level.

#### E. Data quality assessment

**Fig 2:**
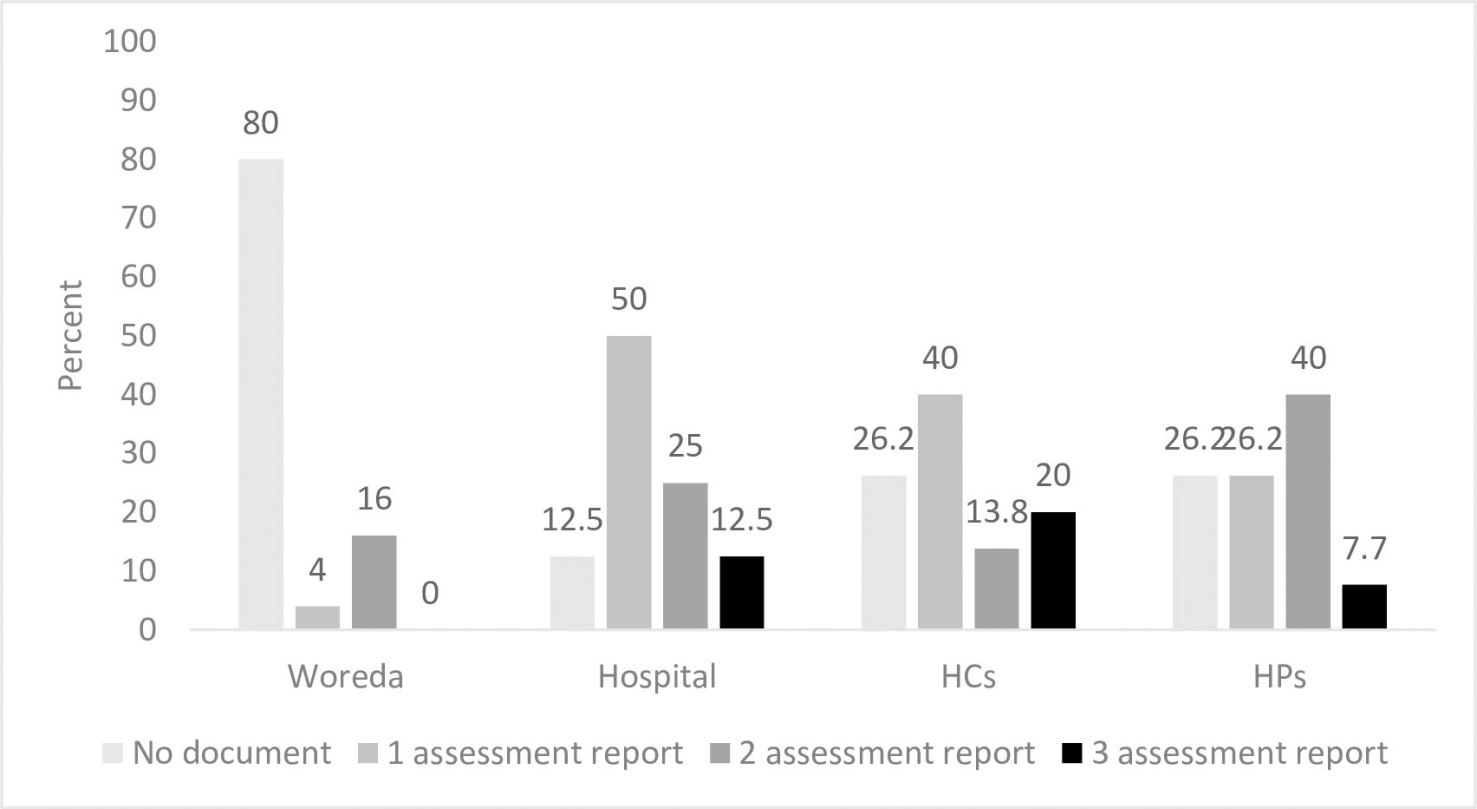
Percent of facilities conducting data quality assessment.

For proper decision making, data in use should be checked for its quality. One of methods of checking data quality is conducting self-data quality assessment routinely, usually monthly for health facilities and quarterly for woredas. Eight (32%) woredas, 53(81.5%) HCs, 8(100%) hospitals and 65 (100%) HPs reported that they routinely conduct data quality assessment. The number of data quality assessments performed (observed) in the quarter was presented in Fig 2.

### Overall summary

**Fig 3:**
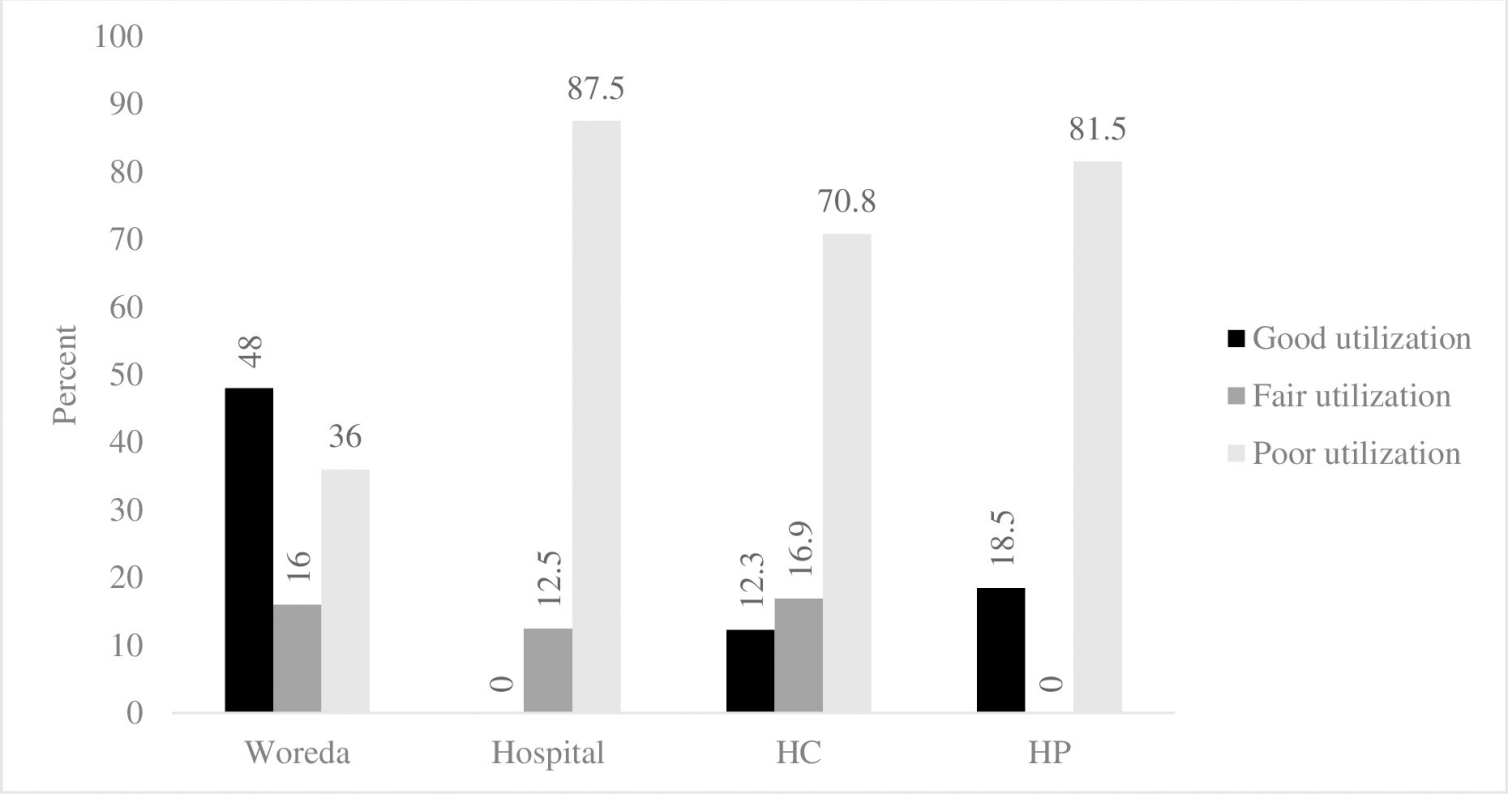
Percentage of public health facilities utilizing HMIS data, SNNPR, 2017.

In general, considering that data utilization comprises problem identification, prioritization, taking action and monitoring actions, overall data utilization score was computed for easy judgment using 8 items from above results. The mean score of woreda, HCs, hospitals and HPs was 55%, 36%, 26% and 29% respectively. Majority of facilities (87.5% hospitals, 81.5% HPs and 70.8% HCs) were poor in data utilization or scored below 50% (Fig 3).

## Discussion

This study assessed data utilization at different levels of public health system in the study area. The results showed that about half of woredas and below one fifth of facilities were utilizing data in a proper way.

The ultimate goal of HMIS is using information to solve problems, which requires building an information use culture over time [14] and improving the health system’s ability to respond to health needs at all levels [22]. In addition, evidence-based decision making has become a benchmark of best practice [23]. To improve the quality of care provided, health systems need to utilize data at all levels, from providers to local and national-level decision-makers [24].

To use data as information, it should be analyzed and interpreted and it is usually done by performance review team (PRT) in the study area. The PRT is a team comprising decision-makers and technical personnel responsible for data analysis and informing decision-makers. The review process is expected to be conducted every month but only 13 (52%) woredas, 17(26.2%) HCs, 2(25%) hospitals and 4 (6.2%) HPs performed it monthly and only 20% woredas, 6.2% HCs, 0% hospitals and 1.5% HPs prepared action plans after review. Summary of 8 items from performance monitoring system showed that majority of facilities (87.5% hospitals, 81.5% HPs and 70.8% HCs) were poor in data utilization (Fig 3). Even though Ethiopia has been implementing standardized HMIS for data capturing, reporting, analyzing and use, lower structures are not effectively using it. This showed that designing and implementing the information system may not realize the information utilization goal as it was noted in the case of Malawi HMIS which showed no matter how good the design of an information system, yet very little improvement has been noted in use of information in rationalizing decisions [18].

Weak HMIS are a critical challenge to reaching the health-related goals because health systems performance cannot be adequately monitored [25]. Improving access to and use of data for decision-making, particularly at subnational levels was reported as significant potential to health system strengthening [26]. Studies including those conducted in developed countries reported that even though study participants desire data use, a relatively small number practice it in local health departments associating it with leadership, workforce capacity (number and skills), resources, funding and program mandates, political support, and access to data and program models suitable to community conditions[27]. And these factors that influence data quality and use are summarized in to technical, behavioral and organizational factors [28, 29].

Most LMICs health system decentralization has increased the decision-making responsibility of sub-national management teams [24]. A literature review done to assess data quality and use in LMICs showed that information use is low and indicated that individual training efforts focus primarily on imparting data analysis skills [12]. Studies done in Nigeria [30] and Tanzania [31] also reported low information use adding technical skill gap as challenge. And limited resources, time constraints, and negative attitudes (or resistance) are some of barriers to implementing supports for evidence-informed decision-making [32].

Even though studies assessing data use were done in Ethiopia, it was difficult to compare levels of data use with current study as focus of measurement was different among studies. One of studies [33] focused at individual level HMIS data utilization and reported good level of use while the other [34] focused on data presentation and reported low level of utilization.

In addition to decentralized decision-making, implementation research done in Ghana reported that several district action plans offer a statistical basis to justify program interventions and funding requests [22]. In resource limited setting like the study region, local evidence based problem identification and prioritization are critical. So, we believe that lower health system should be empowered to ensure data use. Engaging frontline staff and managers in improving data collection and its use is highlighted as important way in improving HMIS [25].

A study reported that facilitating roles that actively promote research use within the organization, establishing ties to researchers and opinion leaders outside the organization, a technical infrastructure that provides access to research evidence (like databases) and participation in training programs to enhance staff's capacity facilitate evidence-informed decision-making [35]. Another study demonstrated the potential for improving evidence-based decision-making capacity among local health department practitioners using a train-the-trainer approach involving diverse partners [36].

Even though we measured data use considering key components of monitoring and evaluation system for evidence-based decision-making, the study was limited in linking data use to level of performance; that is we did not correlate whether those institutions fulfilling our measurement criteria were performing better or not.

## Conclusions

Only about half of woredas and below one-fifth of health facilities were utilizing HMIS data and a lot to move to catch-up country’s information revolution plan. Lower health care systems should be supported in evidence-based decision-making and progress should be monitored routinely quantitatively and/or qualitatively.

## Lists of abbreviations

HC: Health Center
HMIS: Health Management Information System
HP: Health Post
PRT: Performance Review Team
SNNPR: Southern Nations Nationalities and People’s Region
SPSS: Statistical Package for the Social Sciences
